# Exogenous and Endogenous Hormones in Relation to Glioma in Women: A Meta-analysis of 11 Case-Control Studies

**DOI:** 10.1371/journal.pone.0068695

**Published:** 2013-07-16

**Authors:** Zhen-Yu Qi, Chuan Shao, Xin Zhang, Guo-Zhen Hui, Zhong Wang

**Affiliations:** Department of Neurosurgery, First Affiliated Hospital of Soochow University, Suzhou, Jiangsu, China; UCLA, United States of America

## Abstract

**Background and Objective:**

Previous investigations of glioma risk in women have focused on oral contraceptive (OC), hormone replacement therapy (HRT), and reproductive factors. However, the results of published studies were inconclusive and inconsistent. Thus, a meta-analysis based on published case-control studies was performed to assess the role of exogenous and endogenous hormones factors in glioma risk.

**Methods:**

The PubMed and EMBASE databases were searched without any restrictions on language or publication year. Reference lists from retrieved articles were also reviewed. We included case-control studies reporting relative risks (RRs) with corresponding 95% confidence intervals (CIs) (or data to calculate them) between oral contraceptive (OC) and hormone replacement therapy (HRT) use, reproductive factors and glioma. Random-effects models were used to calculate the summary risk estimates.

**Results:**

Finally, 11 eligible studies with 4860 cases and 14,740 controls were identified. A lower risk of glioma was observed among women who were ever users of exogenous hormones (OC RR = 0.707, 95% CI = 0.604–0.828; HRT: RR = 0.683, 95% CI = 0.577–0.808) compared with never users. An increased glioma risk was associated with older age at menarche (RR = 1.401, 95% CI = 1.052–1.865). No association was observed for menopause status, parous status, age at menopause, or age at first birth and glioma risk.

**Conclusion:**

The results of our study support the hypothesis female sex hormones play a role in the development of glioma in women. Additional studies are warranted to validate the conclusion from this meta-analysis and clarity the underlying mechanisms.

## Introduction

Glioma is the most common type of central nervous system tumor, which accounts for more than 70% of cases [1]. Despite decades of research, the etiology of glioma remains unclear. To date, exposure to high doses of ionizing radiation and certain rare genetic syndromes are the only well-established risk factors for glioma [2]. However, there is some evidence implying that sex hormones influence the development and growth of glioma. The incidence of glioma is about 1.5 or 2 fold higher in men than in women, but the sex difference emerges in early adolescence, reaches a maximum around the age at menopause and decreases thereafter [3]–[5]. Furthermore, both animal and biological experiments reinforced the epidemiological data. In vitro studies, people found that steroid hormone receptors are expressed in both normal and glioma cells [6]–[8], and that estrogens can inhibit proliferation of glioma cells as well as induce cell death [9], [10]. In animal experiments, male athymic mice and nude mat transplanted with human glioblastoma cells had bigger tumors, a shorter latency period and lower survival rates compared to females [11]–[13]. To determine to what extent hormonal and reproductive factors influence the risk of glioma in women, a number of studies have assessed the relationship between glioma and female-specific risk factors. Given the inconsistency of the existing literature and the insufficient statistical power of primary studies, a meta-analysis of case-control studies was conducted to derive the most precise estimation.

## Materials and Methods

### Publication Search

We searched the PubMed and EMBASE databases, using the terms “(glioma OR brain cancer OR brain neoplasms OR brain tumor) AND (reproductive factors OR menstrual factors OR age at menarche OR menarche OR menstruation OR parity OR gravidity OR pregnancy OR breastfeeding OR miscarriage OR abortion OR fertility OR age at first birth OR age at menopause OR menopausal status OR estrogens OR sex hormones OR ovariectomy OR oophorectomy OR hysterectomy OR sex differences OR exogenous hormones OR exogenous hormones use OR oral contraceptives (OC) OR hormone replacement therapy (HRT) OR menopausal hormone therapy OR climacteric OR reproductive history) AND (risk assessment OR risk OR risk factors)”. The citations of the identified articles were also screened for additional studies. We neither imposed any limitations on language or publication year nor sent e-mails to the corresponding authors of included studies to retrieve the original data. The latest search was performed on October 1, 2012.

### Inclusion Criteria

Studies were considered eligible if the studies met the following inclusion criteria: (1) a case-control study; (2) tested the association between OC, HRT, reproductive factors and glioma risk; (3) provided estimates of relative risk with corresponding 95% CIs (or data to calculate them); (4) in case of multiple reports of the same trial, we selected the most recent publication with the largest number of subjects.

### Data Extraction

Two authors carefully and independently extracted the following data from each available study: the first author’s last name, publication year, country in which conducted, study period, age of subjects, study design, size of the study population (case/control), proxy interview (yes/no), data collection, exposure variables and categories. Any disagreements were resolved by discussion.

### Assessment of Methodological Quality

The Newcastle-Ottawa Scale (NOS), a validated modality for evaluating observational and non-randomized studies, was used to assess the quality of included studies. This measure assesses aspects of methodology in observational studies related to study quality, including selection, comparability and exposure or outcome. The NOS ranges from zero to nine stars and the studies with ≥6 * were considered to be of relatively higher quality.

### Statistical Analysis

The STATA software, version 11.0 (STATA Corporation, College Station, TX, USA) was used to compute pooled RRs and 95% CIs, generate forest plots, determine whether there was a statistical association, evaluate heterogeneity, perform a sensitivity analysis, and investigate publication bias [14].

The RR was used as the measure of association across studies. Because glioma is a rare disease, ORs (odds ratios) were deemed equivalent to RRs. Therefore, we reported all results as relative risk for simplicity. The most-adjusted risk estimates were retrieved and used in the meta-analyses; however, when unavailable, unadjusted risk estimates were used. The unadjusted risk estimates were extracted directly from the article or computed from the exposure distributions for cases and controls given in the papers. The relative risks and corresponding standard errors (derived from the CIs) from individual studies were transformed to their natural logarithms to stabilize the variances and to normalize the distributions. Heterogeneity between studies was evaluated by Cochran’s Q statistic and heterogeneity was considered significant when P<0.1 [15]. In order to better evaluate the extent of heterogeneity, the I^2^ test was also used. The I^2^ statistic yields results ranged from 0 to 100% (I^2^<25%, low heterogeneity; I^2^ = 25%–50%, moderate heterogeneity; and I^2^>50%, high heterogeneity) [16]. We used the random rather than fixed-effects model to estimate pooled RRs because in the absence of heterogeneity the random-effects model exactly equals the fixed-effects model [17].

Sensitivity analysis and publication bias analysis were performed to assess the stability of the results as previously [18], [19]. Briefly, Egger’s test was performed to assess the publication bias. To reflect the influence of individual datasets to the pooled RRs in our meta-analysis, one study at a time was omitted from the sensitivity analysis.

For all exposure variables and categories, we adopted criteria from original publications. In this meta-analysis, we compared ever users with never users for OC and HRT. For HRT, one study reported the risk estimates of using hormones to treat gynecologic problems [20]. Therefore, the relative risk estimate for ever use versus never use was not included in this meta-analysis.

To assess the effect of reproductive factors, we performed a meta-analysis of the comparison of the highest versus lowest category in each study. For age at menarche and menopause, three studies reported that the oldest age at menopause was compared to the second tertile [21]–[23]; hence, adjusted RRs could not be pooled with others comparing the highest versus lowest categories. In order to standardize the direction of comparisons, we calculated the crude ORs comparing the oldest age group to the youngest group and used them instead. For age at first birth, two studies [22], [24] used nulliparous women as the reference group, whereas the other four studies [21], [25]–[27] used a parous comparison group. To make the standards of comparisons unified, we also calculated the crude ORs for the oldest age group to the youngest age group among parous women and used them to calculate the pooled RR. Due to the limited number of eligible studies, the dose-response analysis and time dependent evaluations were not performed.

## Results

### Literature Search

**Figure 1:**
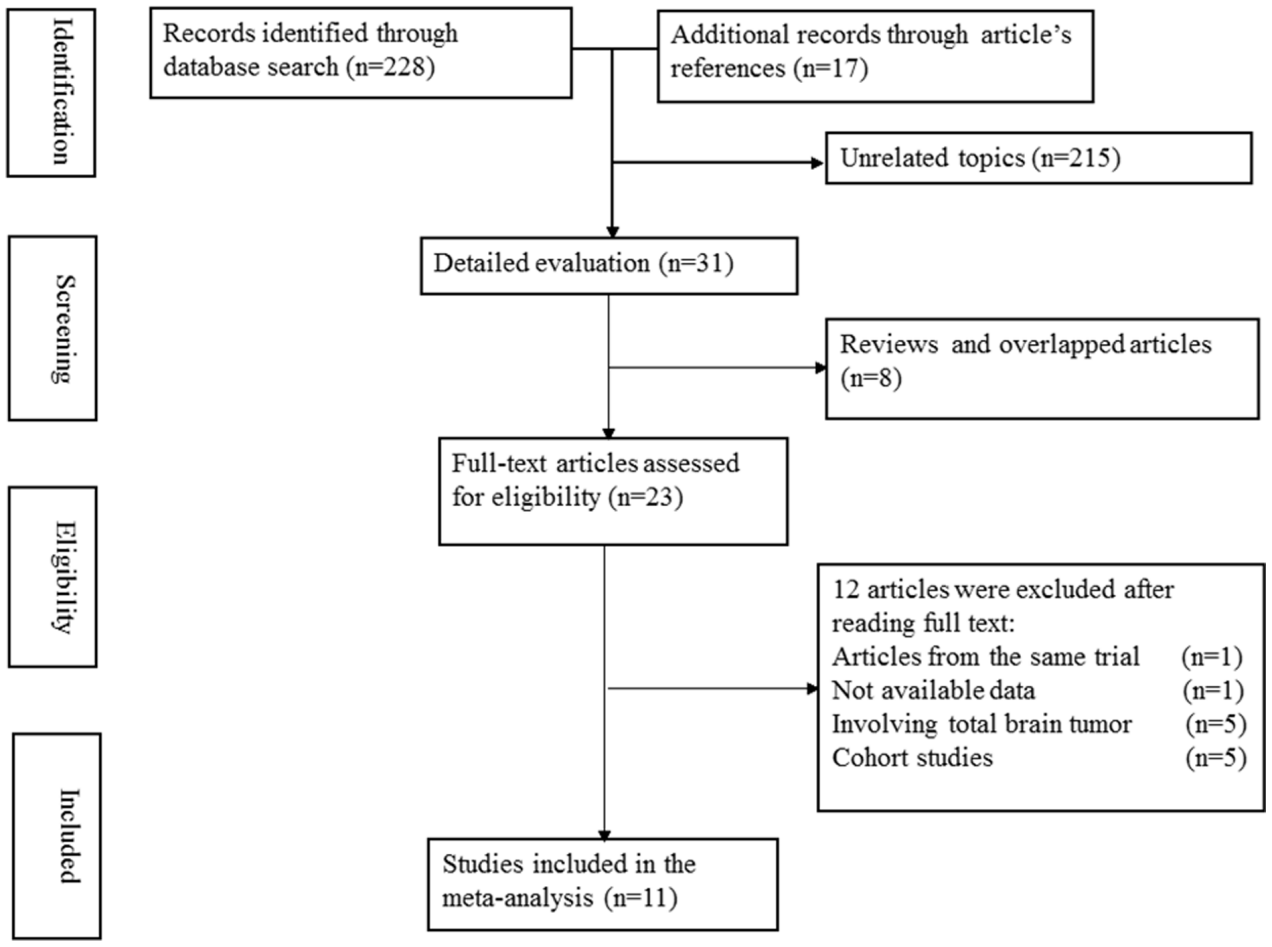
Flow diagram of the study selection process.

Fig. 1 shows a flow diagram of the selection process for relevant studies. A total of 245 articles were initially identified; 228 records were identified in the PubMed and EMBASE databases, and 17 articles which may be related to the topic, were found in article reference lists. Of the 245 articles, 23 records with full text that met the inclusion criteria were assessed. Two articles were from the same trial [28], [29], so only the most recent article was included [29]. One article did not have available data [30], five articles investigated all types of brain tumors in their subjects [31]–[35], and five articles are cohort studies [36]–[40]. Thus, a final total of 11 studies published from 1990 to 2011 were included in this meta-analysis [20]–[27], [29], [41]–[42].

### Characteristics of the Retrieved Studies

Table 1 summarizes the characteristics of included studies. Studies were conducted in the United States, Australia, Swedish, France, Canada, Finland, Denmark Germany, Norway, and the United Kingdom. Of those, 9 studies were population-based [20]–[22], [25]–[27], [29], [41], [42], one was hospital-based [24], and one was mixed of population-based and hospital-based [23]. Studies varied in size, including 67–1657 for cases, and 59–8225 for controls. All cases were histologically confirmed. Controls were randomized. Data were collected by questionnaire, phone interview, in person interview, and medical records from Fertility Registry and Cancer Registry. Concerning the definition of exogenous female hormone, datum was ascertained by asking whether the subjects had ever used OC or HRT. Among these studies, only one study reported women with initial exogenous hormone use within 1 year of the reference date were counted as unexposed to HRT [24]. The others did not take into account the date of exposure occurring before the date of diagnosis (or the date of interview for controls). With regard to the definition of menopausal status, five studies had a detailed description [20]–[22], [24], [27]. The definitions of postmenopausal age differed in each study. The most common is that a woman was defined as postmenopausal if she had her last menstrual period or a bilateral oophorectomy at least one year before the reference date. However, women who reported hysterectomy either with or without bilateral oophorectomy, who had missing or incomplete information on menstruation, or who indicated use of exogenous hormones or intrauterine device while still menstruating were also defined as postmenopausal if they were 50 or 55 years of age or older in some study. For other reproductive factors, no detailed information was reported. To evaluate the methodological qualities of the included studies, NOS was used in our meta-analysis. The results of assessment of methodological quality are shown in Table S1.

**Table 1:** Characteristics of the eleven case-control studies included in the meta-analysis.

First author, Publication year	Country in which conducted	Study period	Age (years)	Study design	Cases/Controls	Proxy interview	Data-collection	Exposure variables and categories
Hochberg, 1990	USA	1977–1981	15–81	PCC	67/59	Yes	Questionnaire or phone interview	OC, Birth order, Mother’s age at birth, Age at menarche.
Cantor, 1993	USA	1984–1989	40–85	PCC	169/821	Yes	Questionnaire or phone interview	Age at first birth, Pregnancy, Parity, First-born.
Cicuttini, 1997	Australia	1987–1991	20–70	PCC	166/170	Yes	In-person interview	Pregnancy, Hysterectomy, Menopausal status.
Lambe, 1997	Swedish	1958–1990	≥15	NCC	1657/8225	NO	Data recorded in Fertility Registry and the Swedish Cancer Registry	Pregnancy, Number of births, Age at first birth.
Schlehofer, 1999	USA, Australia,France, Canada,Germany, Sweden	1980–1991	20–80	PCC	531/933	Yes	Questionnaire or in-person interview	Menopausal status, steroid hormones use.
Huang, 2004	USA	1995–1997	18–80	PCC	341/527	Yes	In-person interview	OC, HRT, Age at menarche, first birth and last birth, Number of live births, Breast Feeding, Menstruation months, Type of menopause.
Hatch, 2005	USA	1994–1998	≥15	HCC	212/436	Yes	Questionnaire or in-person interview	Age at menarche, first birth, and menopause, Ever pregnant, Ever had live birth, Number of live birth, Menopausal status, Type of menopause, Breast Feeding, Bilateral oophorectomy.
Wigertz, 2006	Sweden	2000–2002	20–69	PCC	115/323	Yes	In-person interview, phone interview, or questionnaire	OC, other contraceptives, HRT given gynecologic problems, HRT.
Wigertz, 2008	Sweden, Finland, Denmark, Norway,UK	2000–2004	18–69	PCC	626/1774	Yes	In-person interview or phone interview	Age at menarche and menopause, Pregnant, Menopausal status, Ever had live birth, No of pregnancies leading to a live birth, Breast feeding.
Felini, 2009	USA	1991–1994, 1997–1999, 2001–2004	≥20	PCC	619/650	Yes	In-person interview or phone interview	OC, PHT, Parity, number of children, Gravidity, Age at first birth and menopause, Menopausal type, Menstruation years.
Wang, 2011	USA	1993–2001	18–80	PCC/HCC	357/822	Yes	Questionnaire or in-person interview	OC, MRT, Age at menopause and menarche, Menopause status.

UK, United Kingdom; PCC, population-based case-control study; HCC, hospital-based case-control study; NCC, nested case-control study; PHT, postmenopausal hormone use; MRT, Menopausal hormone therapy use.

### Quantitative Synthesis

#### OC use

**Figure 2:**
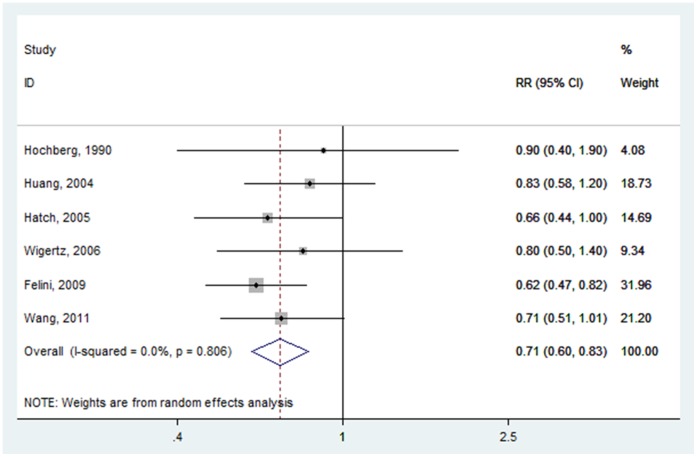
Forest plot of OC use (ever vs. never) and glioma risk.

Risk estimates for OC ever versus never use were reported in six studies [20]–[24], [41]. The overall pooled RR of glioma for ever users versus never users of OC was 0.707 (95% CI: 0.604–0.828) (Fig. 2), with low heterogeneity (P = 0.806, I^2^ = 0.0%). In Hochberg and colleagues’ research, it is not entirely clear that all subjects in this study are female. Therefore, we excluded this study and the cumulative risk estimate was 0.700 (95% CI: 0.596–0.822) (Fig. S1). The pooled RR was not materially altered, suggesting our result was robust and reliable.

#### HRT use

**Figure 3:**
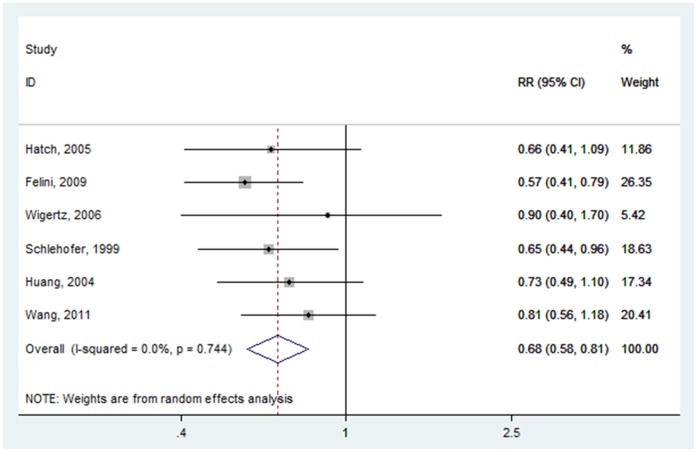
Forest plot of HRT (ever vs. never) and glioma risk.

For HRT, six studies were included in this meta-analysis [20]–[24], [29]. The cumulative risk estimates for ever users versus never users of HRT was 0.683 (95% CI: 0.577–0.808) (Fig. 3), with low heterogeneity (P = 0.744, I^2^ = 0.0%).

#### Menopausal status

**Figure 4:**
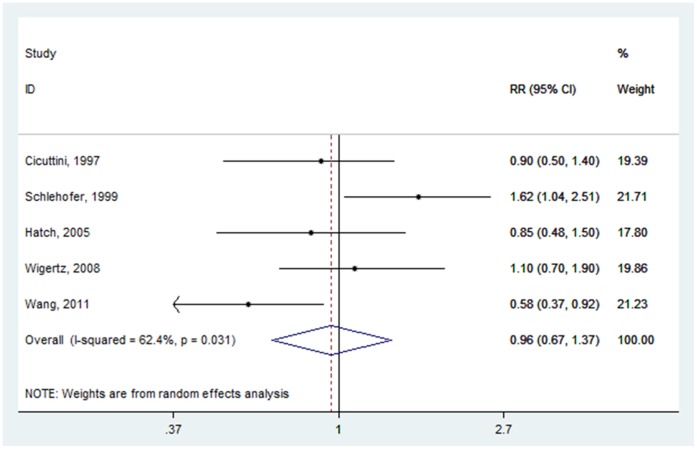
Forest plot of menopausal status (postmenopausal vs. premenopausal) and glioma risk.

Associations of glioma risk with menopausal status were reported in five studies [23], [24], [27], [29], [42]. The summary RR comparing postmenopausal status with premenopausal status was 0.959 (95% CI: 0.670–1.375) (Fig. 4), with high heterogeneity (P = 0.031, I^2^ = 62.4%).

#### Parous status

**Figure 5:**
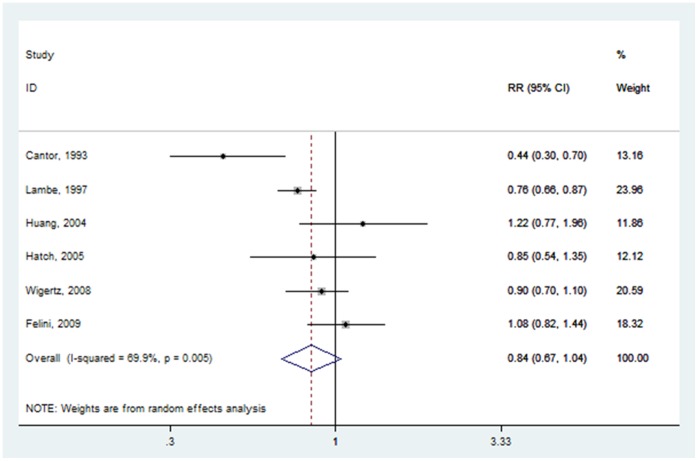
Forest plot of parous status (parous vs. nulliparous) and glioma risk.

Six studies analyzed the role of pregnancy status on glioma risk [21], [22], [24], [25]–[27]. The pooled RR for parous versus nulliparous was 0.837 (95% CI: 0.674–1.040) (Fig. 5), with high heterogeneity (P = 0.005, I^2^ = 69.9%).

#### Age at menarche

**Figure 6:**
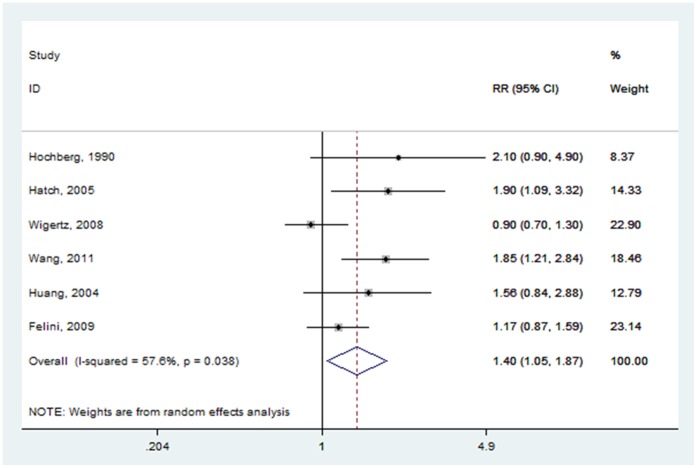
Forest plot of age at menarche (oldest vs. youngest) and glioma risk.

Six studies examined the association of glioma and age at menarche [21]–[24], [27], [41]. Two crude RRs [21], [22] were calculated according to the number of cases and controls. The pooled RR for the oldest age group versus the youngest age group was 1.401(95% CI: 1.052–1.865) (Fig. 6), with high heterogeneity (P = 0.038, I^2^ = 57.6%).

#### Age at menopause

**Figure 7:**
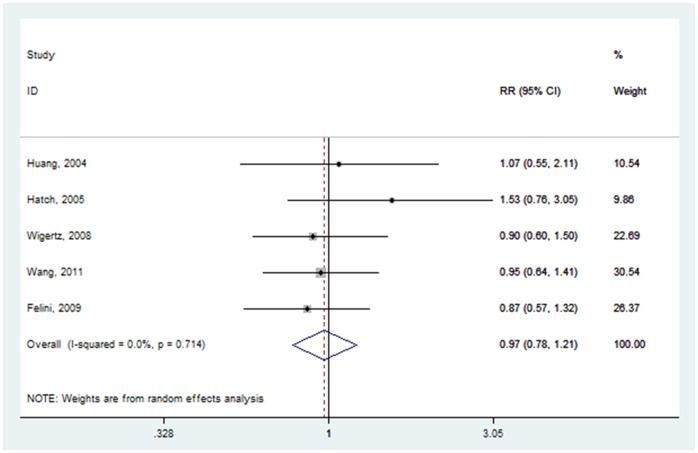
Forest plot of age at menopause (oldest vs. youngest) and glioma risk.

Five articles provided information on glioma risk with age at menopause [21]–[24], [27], of which two crude RRs [22], [23] were calculated based on the number of cases and controls. The pooled RR for the oldest age group versus the youngest age group was 0.972 (95% CI: 0.782–1.209) (Fig. 7), with low heterogeneity (P = 0.714, I^2^ = 0.0%).

#### Age at first birth

**Figure 8:**
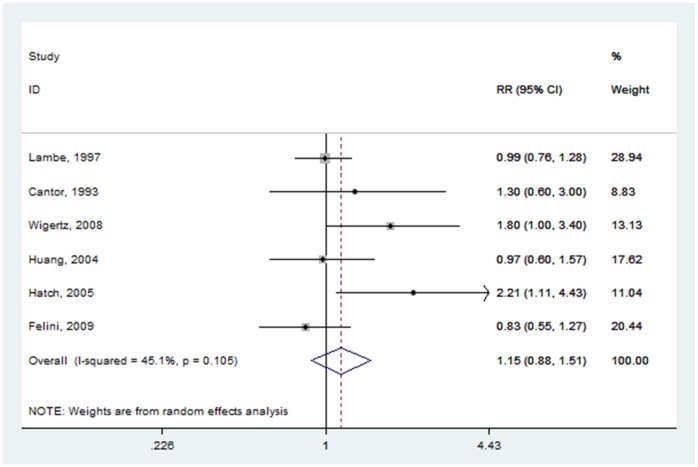
Forest plot of age at first birth (oldest vs. youngest) and glioma risk.

The effect of age at first birth was examined by six studies [21], [22], [24]–[27], of which two crude RRs [22], [24] were calculated based on the original data. The summary RR for the oldest age group versus the youngest age group at first birth was 1.153 (95% CI: 0.879–1.513) (Fig. 8), with moderate heterogeneity (P = 0.105, I^2^ = 45.1%).

All results of the meta-analysis were also presented in Table 2.

**Table 2:** Summary relative estimates for glioma in women.

	Exposure Categories			Heterogeneity		
Risk factors	Highest(min to mix) vs. Lowest(minto mix)	Number of studies(Reference)	Pooled RR(95% CI)	P_Q_	I^2^	P_Egger’s_
OC	Ever vs. Never	6 (20–24, 41)	0.707(0.604–0.828)	0.806	0.0%	0.144
HRT	Ever vs. Never	6 (20–24, 29)	0.683(0.577–0.808)	0.744	0.0%	0.252
Menopausal status	Postmenopausal vs. Premenopausal	5 (23, 24, 27, 29, 42)	0.959(0.670–1.375)	0.031	62.4%	0.699
Parous status	Parous vs. nulliparous	6 (21, 22, 24, 25–27)	0.837(0.674–1.040)	0.005	69.9%	0.779
Age at menarche	Oldest (≥14) vs. Youngest(≤11 to ≤12)	6 (21–24, 27, 41)	1.401(1.052–1.865)	0.038	57.6%	0.076
Age at menopause	Oldest(≥50 to ≥53) vs. Youngest(≤40 to ≤50)	5 (21–24, 27)	0.972(0.782–1.209)	0.714	0.0%	0.093
Age at first birth	Oldest(≥30 to ≥35) vs. Youngest(≤20 to ≤25)	6 (21, 22, 24–27)	1.153(0.879–1.513)	0.105	45.1%	0.146

### Sensitivity Analysis

**Figure 9:**
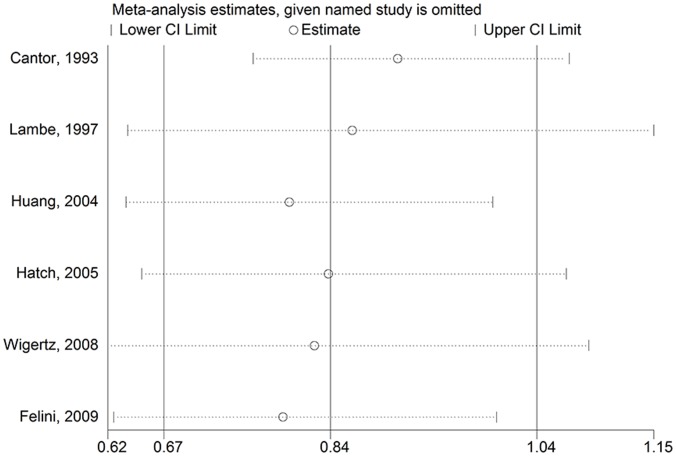
Sensitivity analyses for parous status (parous vs. nulliparous) and glioma risk.

**Figure 10:**
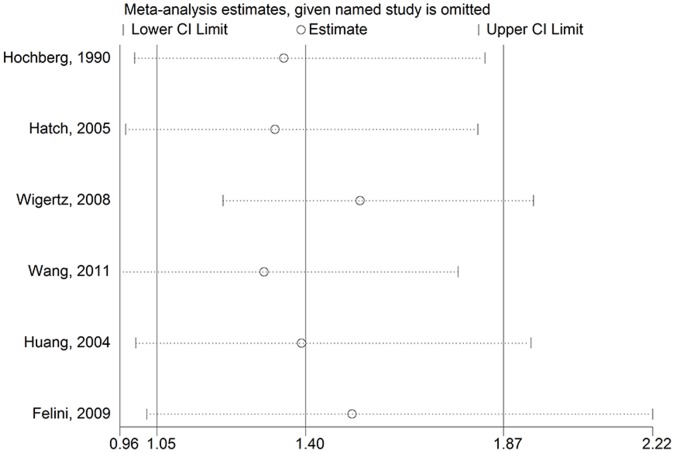
Sensitivity analyses for age at menarche (oldest vs. youngest) and glioma risk.

In the sensitivity analysis, we excluded one single study at a time to investigate the influence of each single study on the overall risk estimate. For parous status, the combined RR for parous versus nulliparous was 0.797 (95% CI: 0.637–0.997) (Fig. 9) after excluding the study [21]. For age at menarche, no significant associations were observed after omitting Wang and colleagues’ research [23] and Hatch and colleagues [21]. The pooled RRs were 1.329 (95% CI: 0.978–1.805) (Fig. 10) for Hatch and colleagues [21] and 1.303 (95% CI: 0.965–1.759) (Fig. 10) for Wang and colleagues’ research [23]. For other risk factors comparisons, the corresponding pooled RRs were not materially altered (data not shown).

### Publication Bias

The results of Egger’s test suggested there was no evidence of publication bias (The results of Egger’s test were presented in Table 2).

## Discussion

To our knowledge, this meta-analysis of eleven published case-control studies is the first of its kind to evaluate the association between glioma risk and exogenous and endogenous hormones. The results of our meta-analysis suggest a decreased risk for ever users of OC or HRT compared with never users, whereas older age at menarche was associated with a statistically increased risk of glioma. However, the risk of glioma seemed to not be influenced by menopausal status, parous status, age at menopause, or age at first birth.

Demonstration of a dose-response relationship in observational studies lends support to a suspected causal relation-ship between exposure and disease. However, we were unable to perform the dose-response analysis and time dependent evaluations because the original studies did not provide the required data. Few studies evaluated the patterns of OC or HRT use in women with glioma. Moreover, several previous studies had suggested progesterone, estrogen, and androgen receptors are expressed in glioma in various degrees [6]–[8]. Therefore, further evaluation of exogenous hormone use in women with glioma is needed with particular attention to stratification by hormone composition (i.e., estrogen and/or progesterone), duration of use and age at start of therapy as well as tumor receptor subtype.

In this meta-analysis, we found a decreased risk of glioma in postmenopausal women who were ever users of HRT. However, a large Women’s Health Initiative randomized controlled trial published in 2002 suggests that overall health risks outweighed the benefits from use of estrogen plus progestin among healthy postmenopausal US women [43]. Rossouw et al reported that while HRT decreased the incidence of hip fractures and colorectal cancer, it moreover increased the risk of invasive cancer, coronary heart disease, strokes, and pulmonary embolism. In our study, most of the participants were recruited before 2002. These women may use HRT at doses that are no longer recommended for those recruited after 2002 because of the disease risks listed above. Furthermore, the result of this meta-analysis may be detected by chance because of the limited numbers of case-control studies. Thus, our finding should be interpreted with caution.

Substantial heterogeneity was observed across studies of the associations of menopausal status, parous status, and age at menarche with glioma risk. This is not surprising given the variation in study designs and characteristics of populations between studies. All studies included in this meta-analysis were case-control studies, but some studies used a population based case-control design, while others used a hospital-based case-control design. In addition, our meta-analysis included a nested case-control study. Studies identified in this meta-analysis were performed in different geographic regions, mostly European and North American countries, where people share a variety of genetic backgrounds and lifestyles.

Our study is supported by the following strengths. Because individual studies have insufficient statistical power, our meta-analysis of 11 studies involving a large number of subjects enhanced the power to detect significant associations and provided more reliable estimates. All the original studies used a case-control study design, which greatly reduced the heterogeneity between studies. Moreover, our results are consistent with the experimental, biological, and epidemiological data [3]–[5], [9]–[13].

Despite these advantages, some limitations of the current study should be considered when interpreting our results. First, all the included studies used a case-control study design. Thus, the likelihood of recall and selective biases that are always of concern in case-control studies may be greatly increased. Second, when sensitivity analysis was conducted in all comparisons, we found that the results of two comparisons (parous versus nulliparous, and the oldest age group versus the youngest age group at menarche) were not robust. Third, measurement error associated with exposure should be noted. Almost all studies included in our meta-analysis used a certain percentage of proxy-reporting measures. Hence, the pooled estimation provided less clear results. Fourth, potential publication bias might influence the findings, yet little evidence of publication bias was observed in the present meta-analysis. Finally, because the results of the current meta-analysis mainly involved Western populations, additional research in other populations is warranted to generalize the findings.

In conclusion, a decreased risk of glioma with OC or HRT use was observed, whereas the risk of glioma increased with older age at menarche. Therefore, we supposed that female sex hormones closely relate with the risk of glioma. Further prospective research is warranted to extend this finding by increasing the sample sizes and considering multiple factors.

## Supporting Information

Figure S1 **Forest plot of OC use (ever vs. never) and glioma risk after excluding Hochberg and colleagues’ research.**
(TIF)

Table S1 **Methodological quality of included studies based on the Newcastle–Ottawa Scale for assessing the quality of included studies.**
(DOCX)
