# Supplementary figures and images for: Exogenous and Endogenous Hormones in Relation to Glioma in Women: A Meta-analysis of 11 Case-Control Studies
Source: PLoS One. 2013 Jul 16;8(7):e68695. doi: 10.1371/journal.pone.0068695 (PMC3712935; doi:10.1371/journal.pone.0068695)

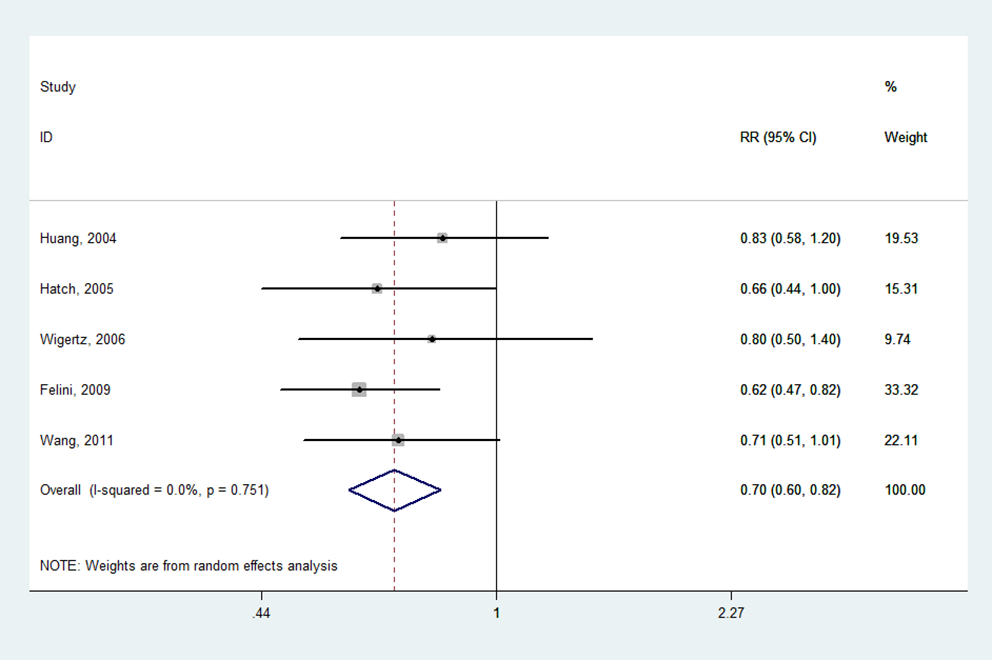

Supplement: Figure S1 — Forest plot of OC use (ever vs. never) and glioma risk after excluding Hochberg and colleagues’ research. (TIF) [file pone.0068695.s001.tif]
