# Supplementary material for: Exogenous and Endogenous Hormones in Relation to Glioma in Women: A Meta-analysis of 11 Case-Control Studies
Source: PLoS One. 2013 Jul 16;8(7):e68695. doi: 10.1371/journal.pone.0068695 (PMC3712935; doi:10.1371/journal.pone.0068695)
Supplement: Table S1 — Methodological quality of included studies based on the Newcastle–Ottawa Scale for assessing the quality of included studies. (DOCX) [file pone.0068695.s002.docx]

Table S1 Methodological quality of included studies based on the Newcastle–Ottawa Scale for assessing the quality of included studies.

| First author,  Publication year | Selection (score) | | | |  | Comparability(score) |  | Exposure (score) | | | Total  Score |
| --- | --- | --- | --- | --- | --- | --- | --- | --- | --- | --- | --- |
|  | Adequate definition of patient cases | Representativeness  of patients cases | Selection  of controls | Definition  of controls |  | Control for  Important factor or  additional factor |  | Ascertainment  of exposure  (blinding) | Same method of ascertainment for participants | Non-response  rate |  |
| Hochberg, 1990 | 1 | 0 | 1 | 1 |  | 2 |  | 0 | 1 | 0 | 6 |
| Cantor, 1993 | 1 | 1 | 1 | 1 |  | 2 |  | 0 | 1 | 0 | 7 |
| Cicuttini, 1997 | 1 | 1 | 1 | 1 |  | 2 |  | 0 | 1 | 0 | 7 |
| Lambe, 1997 | 1 | 1 | 1 | 1 |  | 2 |  | 1 | 1 | 0 | 8 |
| Schlehofer, 1999 | 1 | 1 | 1 | 1 |  | 2 |  | 0 | 1 | 0 | 7 |
| Huang, 2004 | 1 | 1 | 1 | 1 |  | 2 |  | 0 | 1 | 1 | 8 |
| Hatch, 2005 | 1 | 1 | 0 | 1 |  | 2 |  | 0 | 1 | 0 | 6 |
| Wigertz, 2006 | 1 | 1 | 1 | 1 |  | 2 |  | 0 | 1 | 0 | 7 |
| Wigertz, 2008 | 1 | 1 | 1 | 1 |  | 2 |  | 0 | 1 | 0 | 7 |
| Felini 2009 | 1 | 1 | 1 | 1 |  | 2 |  | 0 | 1 | 1 | 8 |
| Wang, 2011 | 1 | 1 | 0 | 1 |  | 2 |  | 0 | 1 | 0 | 6 |
